# Dual laser-assisted hatching: an effective technique to salvage low-grade cleavage-stage embryos and harvest day 7 blastocysts

**DOI:** 10.1007/s10103-023-03898-9

**Published:** 2023-09-30

**Authors:** Weihai Xu, Yiqi Yu, Shishi Li

**Affiliations:** Center for Reproductive Medicine, Department of Reproductive Endocrinology, Zhejiang Provincial People’s Hospital, Affiliated People’s Hospital, Hangzhou Medical College, 158 Shangtang Road, Hangzhou, 310014 People’s Republic of China

**Keywords:** In vitro fertilization, Embryo, Blastocyst, Laser-assisted hatching, Morula

## Abstract

To investigate whether repeating laser-assisted hatching (LAH) procedure on day 6 low-grade cleavage-stage embryos (LGCEs) helps blastulation. A total of 579 cycles with LGCEs from 2019 to 2022 was retrospectively reviewed. In 323 cycles, single LAH producing small holes (10 μm) was performed on LGCEs on day 4 (D4-LAH). In 256 cycles with persistent LGCEs despite D4-LAH, a repeat LAH procedure was performed on day 6 (Dual-LAH) with a bigger hole (30 μm). We compared day 7 blastocyst formation rate, usable blastocyst rate, and good grade blastocyst rate from these day 6 LGCEs between the two groups. Compared to the D4-LAH group, the Dual-LAH group had both higher day 7 blastocyst formation rate (9.4% vs. 3.0%, *p* < 0.001) and higher day 7 usable blastocyst rates (7.4% vs. 2.1%, *p* < 0.001). For persistent LGCEs despite single LAH, performing a repeat LAH on day 6 increased day 7 blastocyst formation rate.

## Introduction

Zona pellucida (ZP), composed of glycoproteins, is the outer matrix of ovum or embryo [1]. It is an indispensable component in natural reproduction. To achieve successful implantation onto the endometrium, an embryo needs to hatch from the zona pellucida at the stage of blastocyst. Therefore, failed hatching process is believed to be one of the causes for implantation failure [2]. Based on this, artificial removal of the whole or part of zona pellucida was explored to increase the blastocyst hatching rate so as to improve implantation outcomes [3]. Yet related clinical data is still lacking [1, 4]. In fact, how to obtain embryos and develop into blastocysts is one of the key factors in IVF/ICSI cycles [5, 6].

There are 17.5% population suffers from infertility and requires reproductive assistance in the world [7]. Moreover, as much as 10% of in vitro fertilization (IVF) cycles end up with nothing but low-grade cleavage-stage embryos (LGCEs), it means a huge number of IVF patients unable to obtain high-quality embryos for pregnancy. Compared to high-quality embryos, LGCEs exhibit lower potential to develop into blastocyst, which reduces clinical pregnancy rate. The most common manifestations of LGCEs include delayed cell division, unevenness of blastomeres, and fragmentation. LGCEs usually have not yet formed blastocysts at day 6 due to poor developmental potential, and the blastocyst formation rate (BFR) of LGCEs with traditional embryo culture technique is unsatisfactory [8]. Recent studies showed that LGCEs undergoing D4-LAH achieved higher BFR [9]. However, nearly half of the LGCEs still failed to develop after LAH. It is of great clinical value to explore more effective ways to promote LGCE development and blastocyst formation so as to harvest maximal usable embryos.

Based on previous practice, very few embryos form blastocysts on day 7 in vitro, and day 7 blastocysts are associated with low pregnancy rate. Nevertheless, transferring day 7 blastocysts still increases cumulative live births [10–12], especially for patients with only LGCEs available. Therefore, culturing LGCE to obtain more blastocysts not only increases the live birth rate [13] but also eases mental and economic burden for infertile couples. There have been some reported methods that help promote the formation of blastocysts, such as utilizing a low-lactic acid culture medium [14], decreasing the partial pressure of oxygen in the culture environment [15], and adding melatonin to the culture medium [16]. But culturing day 7 blastocysts remains a challenge. Our earlier studies have demonstrated that D4-LAH reduced the probability of undesirable contact between trophoblasts and harmful substances by promoting early hatching through the ZP [9]. But when the embryos were cultured to day 6, blastomere degeneration and fragmentation accumulation recurred. Moreover, the “artificial channel” established by LAH may block again. So in this study, we tried to perform LAH twice on day 4 and day 6 respectively to further open up zona pellucida in LGCEs which failed blastocyst formation on D6. This new method may direct embryos to blastocyst formation and better development, thereby helping to obtain more treatment opportunities for infertile couples.

## Materials and methods

### Study participants

IVF cycles with blastocyst culture process in Zhejiang People’s Hospital from January 2019 to October 2022 were analyzed retrospectively. The inclusion criteria were as follows: (1) fresh embryos, (2) female age of 42 years or less, and (3) at least one of the cultured embryos was LGCE. The exclusion criteria were as follows: (1) numerical or structural abnormalities of chromosomes in either one of the couples, (2) rescue intracytoplasmic sperm injection (rescue ICSI), (3) sperm or oocyte donation cycles, and (4) extremely low-grade cleavage-stage embryos with less than four blastomeres or more than 50% fragmentation. Five hundred seventy-nine cycles were recruited and divided into two groups: the D4-LAH group in which LGCEs received a single LAH procedure on day 4, and the Dual-LAH group in which persistent LGCEs received a repeat LAH procedure on day 6 on top of the day 4 LAH. This study was approved by the Ethics Committee of Zhejiang Provincial People’s Hospital (approval number: SZ2017011).

### Controlled ovarian hyperstimulation (COH) protocols and embryo culture

The COH protocols included the antagonist protocol, long agonist protocol, mild stimulation protocol, progestin-primed ovarian stimulation (PPOS), and ultra-long agonist protocol [17]. Recombinant human chorionic gonadotropin (r-hCG) (Merck Serono, Germany), triptorelin (Ferring, Germany), or a combination of both r-hCG and triptorelin was used for triggering to promote oocyte maturation when the diameters of at least two dominant follicles reached 18 mm. Oocytes were retrieved 36 to 38 h thereafter. IVF or ICSI was performed 39 to 40 h after ovulation induction, and the pronuclear (PN) check for fertilization was performed 16 to 18 h later. The culture medium used in the study were G-IVF (Vitrolife Sweden AB, Sweden), G-1 (Vitrolife Sweden AB, Sweden), and G-2 (Vitrolife Sweden AB, Sweden). The incubator conditions were 6% CO_2_, 5% O_2_, and 89% N_2_. The oocytes were placed in G-IVF for fertilization and transferred to G-1 after 16 to 18 h later. Every embryo was cultured to day 3 and scored according to ASEBIR consensus [18] by two embryologists. Grade A and grade B embryos were good-quality embryos: grade A: 7–8 symmetrical blastomeres with fragmentation ≤ 10%, no multinucleation; grade B: 7–8 symmetrical blastomeres with fragmentation > 11% and ≤ 25%, or ≥ 9 cells with symmetry and ≤ 25% fragmentation. The rest were LGCEs.

### Blastocyst culture and laser-assisted hatching

LGCEs with more than four blastomeres and less than 50% fragmentation were transferred to G-2 blastocyst medium and underwent LAH on day 4 using the Octax Laser Shot System (Vitrolife, Bruckberg, Germany). A 10 μm incision was made on the ZP using a 1480 nm near-infrared diode, with a 2.6 ms laser pulse and 150 J per pulse focusing energy.

Blastocysts were graded according to the Gardner et al. [19] criteria. The stages were determined by the degrees of development and expansion: grades 1–2 being early blastocysts, grade 3 complete blastocysts, grade 4 expanded blastocysts, grade 5 hatching blastocysts, and grade 6 fully hatched blastocysts. The inner cell mass (ICM) was graded as follows: A, plenty ICM cells tightly packed; B, several loosely distributed ICM cells; C, scarce ICM cells. The trophectoderm (TE) was graded as follows: A, plenty TE cells tightly aligned; B, several TE cells loosely distributed; C, scare TE cells. Grade 3BB and above were categorized as good blastocysts. Grade 3 and above blastocysts with a grade C in ICM or TE were suboptimal blastocysts. Both good and suboptimal blastocysts are considered usable blastocysts.

**Fig. 1:**
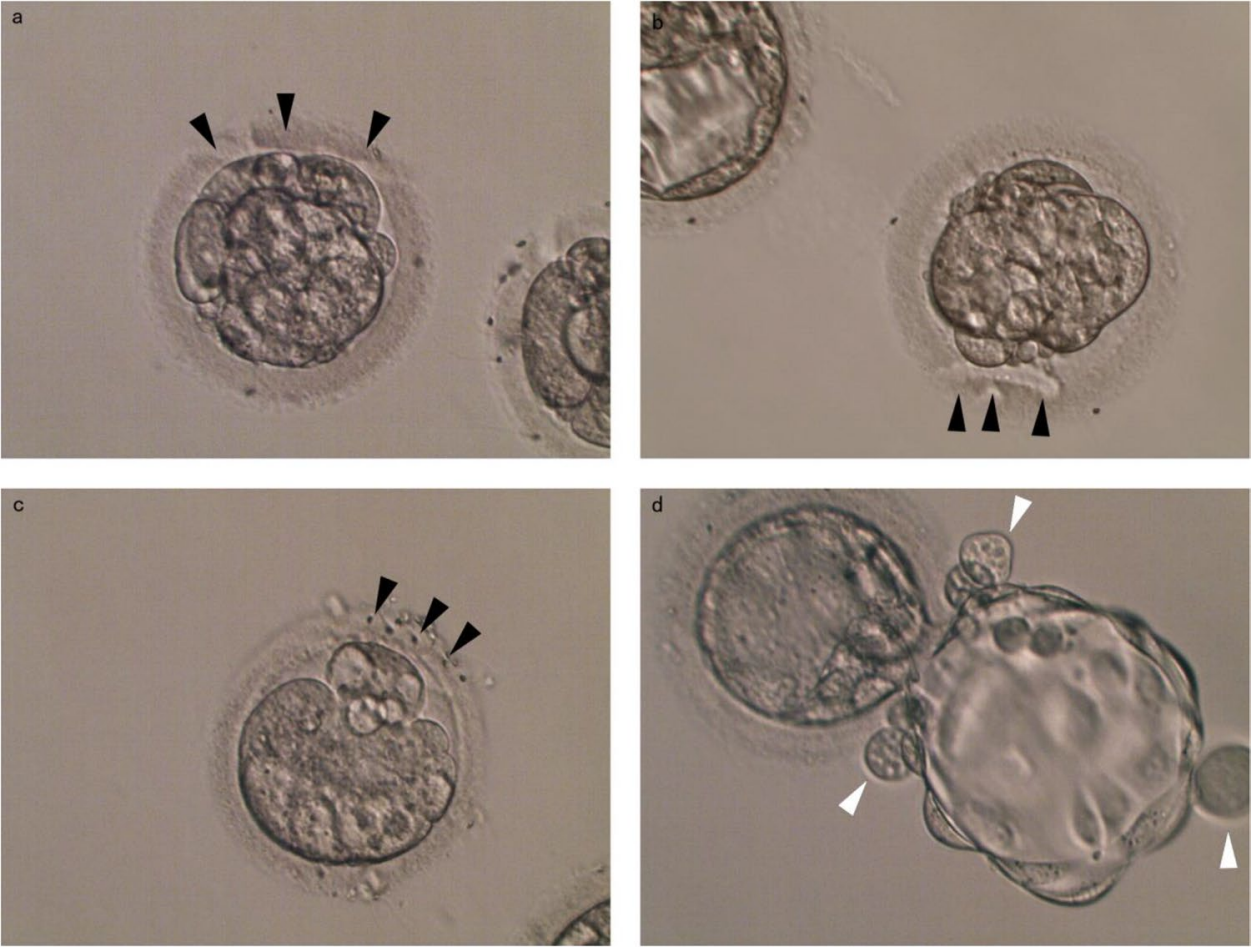
Microscopic pictures of day 6 LGCEs post LAH. The black arrow shows the path of laser drilling. **a** Linear mode. **b** “C”-shaped mode. **c** Laser beam crossed free blastomeres. **d** Fragments and blastomeres are released from ZP after Dual-LAH, as indicated by the white arrow. The images were taken using an inverted microscope with a magnification of 200 times

Blastocysts graded 3BC and above on day 5 were either frozen or used for fresh transfer. The rest were transferred to a fresh G-2 medium for extended culturing. On the morning of day 6, all blastocysts graded 3BC and above were frozen. The remaining embryos received a repeat LAH. Repeat LAH was positioned and operated in a linear or “C” shape based on the spatial position of the blastomeres. A portion of ZP with a diameter of more than 30 μm was dissected locating near fragmentation aggregation site and away from the blastomeres. As for formed morula, LAH could be performed on the excluded blastomeres, as shown in Fig. 1a and b. New batch of usable blastocysts were frozen on day 7. The number of day 7 blastocysts did not include those already formed blastocysts by day 6 but were still being cultured.

### Statistical analysis

Statistical analysis was carried out using SPSS 26.0 software. The main outcomes included BFR, usable blastocyst rate, and good blastocyst rate harvested up to day 7. Normally distributed data were presented as mean ± standard deviation (SD), and the variables between groups were compared using *t*-tests and chi-squared/*F*-tests. Association between Dual-LAH and formation of day 7 blastocysts, day 7 usable blastocysts, and day 7 good blastocysts was examined by log-binomial regression with generalized estimating equations (GEE), adjusted for female age, infertility duration, causes of infertility, primary/secondary infertility, body mass index (BMI), anti-Müllerian hormone (AMH), COH protocols, and the number of oocytes. A two-sided *p*-value of < 0.05 was considered statistically significant.

## Results

### Comparison of the basic characteristics of the two groups

A total of 579 cycles, including 323 cycles in the D4-LAH group and 256 cycles in the Dual-LAH group, were included in this study. Differences in female age, BMI, AMH, causes of infertility, primary/secondary infertility, and infertility duration were not statistically different. There was no significant difference in the number of oocytes, fertilization protocols, fertilization rate, and good embryo rate. However, there was significant difference in the COH protocol compositions between the two groups (*p* = 0.005) (Table 1).

**Table 1:** Basic characteristics of the population

	Dual-LAH group	D4-LAH group	*p*
Cycles	256	323	
Female age (years)	32.1 ± 3.8	32.2 ± 4.6	0.628
Causes of infertility, *n*			
Female factor	99	134	0.631
Male factor	41	52	
Combined causes	97	121	
Idiopathic	19	16	
BMI (kg/m^2^)	21.9 ± 2.9	21.9 ± 3.0	0.841
Primary/secondary infertility	133/123	157/166	0.452
Infertility duration (years)	3.1 ± 2.4	3.3 ± 2.3	0.376
AMH (ng/ml)	3.1 ± 2.2	3.0 ± 2.3	0.628
COH protocols, *n*			
GnRH antagonist protocol	117	188	
Long-agonist protocol	21	33	0.005
Mild stimulation protocol	83	71	
PPOS	23	15	
Ultra-long agonist protocol	12	16	
No. of oocytes	7.9 ± 4.2	8.2 ± 4.6	0.363
Fertilization protocol (IVF/ICSI)	164/92	184/139	0.088
Fertilization rate (%)	70.6 (1427/2021)	69.5 (1849/2660)	0.417
Good embryo rate (%)	48.4 (680/1416)	50.0 (916/1831)	0.181

*COH*, controlled ovarian hyperstimulation

On day 5 and day 6, there was no significant difference in total BFR, usable blastocyst rate, and good blastocyst rate between the two groups. The proportion of morula in the cultured embryos on day 6 was not significantly different between the Dual-LAH group and the D4-LAH group (13.8% vs. 12.9%, *p* > 0.05). The two groups of embryos were comparable up to day 6, shown in Table 2.

**Table 2:** Blastocyst formation in the two groups

	Dual-LAH group	D4-LAH group	*p*
Total no. of LGCEs	736	943	
BFR on day 5 + day 6 (%)	50.8 (374/736)	50.0 (471/943)	0.724
Usable blastocyst rate on day 5 + day 6 (%)	35.2 (259/736)	31.4 (296/943)	0.100
Good blastocyst rate on day 5 + day 6 (%)	11.7 (86/736)	10.8 (102/943)	0.576
No. of LGCEs on day 6	362	472	
Morula on day 6 (%)	13.8 (50/362)	12.9 (61/472)	0.617

### Comparison of the outcomes of day 7 blastocyst between the two groups

A total of 9.0% cycles in the Dual-LAH group obtained blastocysts on day 7, higher than the 2.5% in the D4-LAH group (*p* = 0.001). Compared to the D4-LAH group, the Dual-LAH group also had a higher proportion of cycles with usable blastocysts (7.4% vs. 2.2%; *p* = 0.004). There was no significant difference in the proportion of good blastocyst cycles between the two groups (Table 3).

**Table 3:** Number of cycles with day 7 blastocysts in the two groups

	Dual-LAH group	D4-LAH group	*p*
Total cycles	256	323	
Cycles with at least one day 7 blastocyst formation (%)	9.0 (23/256)	2.5 (8/323)	0.001
Cycles with at least one day 7 usable blastocyst (%)	7.4 (19/256)	2.2 (7/323)	0.004
Cycles with at least one day 7 good blastocyst (%)	1.2 (3/256)	0.6 (2/323)	0.659

There were in total 362 LGCEs in the D4-LAH group and 472 LGCEs in the Dual-LAH group respectively remaining for further culturing on day 6. From these LGCEs, the Dual-LAH group seemed to have a higher BFR (9.4% vs. 3.0%, *p* < 0.001) and usable blastocyst rate (7.4% vs. 2.1%, *p* < 0.001). But there was no statistically significant difference in the good blastocyst rate (4.2% vs. 1.2%, *p* > 0.05) (Table 4). All the day 7 blastocysts in the two groups originated from morulae on day 6.

**Table 4:** Day 7 blastocyst formation in the two groups

	Dual-LAH group	D4-LAH group	*p*
No. of LGCEs on day 6	362	472	
Blastocyst formation rate on day 7 (%)	9.4 (34/362)	3.0 (14/472)	<0.001
Usable blastocyst rate on day 7 (%)	7.4 (27/362)	2.1 (10/472)	<0.001
Good blastocyst rate on day 7 (%)	0.8 (3/362)	0.6 (3/472)	0.744

The proportion of stage 6 day 7 blastocysts in the Dual-LAH group was 79.4% (27/34), higher than that in the D4-LAH group (14.3%, 2/14) (*p* < 0.001). We noted that day 7 grade 6 blastocyst ends to have fewer TE cells. In the Dual-LAH group, some embryos were observed to be expelling fragments and blastomeres from ZP (Fig. 1d).

We then analyzed the correlation between Dual-LAH and the formation of day 7 blastocysts from the remaining LGCEs on day 6, and adjusted for relevant variables. We found that the Dual-LAH correlated with a higher rate of blastulation on day 7 (*p* < 0.001) and usable blastocyst formation on day 7 (*p* < 0.001). Meanwhile, it did not affect good blastocyst formation rate on day 7 (Table 5).

**Table 5:** Multivariate analysis of blastocyst formation on day 7

	OR of Dual-LAH	95% CI		*p*
		Lower	Upper	
Blastocyst count	4.320	2.206	8.461	<0.001
Usable blastocyst count	4.818	2.206	10.521	<0.001
Good blastocyst count	2.060	0.357	11.882	0.419

Adjusted variables: female age, primary/secondary infertility, causes of infertility, infertility duration, BMI, AMH, COH protocol, number of oocytes, and fertilization protocols

## Discussion

In this study, we found that undergoing Dual-LAH on both day 4 and day 6 on LGCEs can increase BFR and usable blastocyst rate on day 7. Simple and practical as it is, Dual-LAH is expected to be a promising technique to make the most value out of LGCEs.

Laboratories in many IVF centers are extending embryo culture to day 7, because day 7 blastocysts, even with their compromised developmental potential, have brought successful pregnancies [20, 21]. A major technical limitation therein is the very low blastocyst formation rate from the remaining day 6 embryos [22, 23]. In this study where all target embryos were LGCEs, the culturing difficulty was even higher and the expectation was even lower. Nevertheless, we achieved a BFR of 9.4% and usable blastocyst rate of 7.4% using the Dual-LAH technique, providing extra opportunities for couples with only LGCEs but strongly yearning for fertility.

We took into consideration the importance of culture medium in day 7 blastocyst formation from LGCEs. Orvieto et al. observed that the majority of embryos that failed to develop into blastocysts were euploid [24], which gave rise to suspicion on other factors that might lead to developmental arrest, such as culture conditions. It has been proven that not changing the embryo culture medium for too long can lead to nutrient deficiencies as well as accumulation of embryotoxic substances [25], resulting in stunted embryo development. This is the reason why we replaced the blastocyst medium on day 5. However, fragments continued to form in day 6 embryos inevitably. Blastomere degeneration and the collection of fragments led to reactive oxygen species (ROS) generation [26], and physical obstruction on intercellular interaction [27], which further disturbed blastocyst formation.

Some studies have also explored the methods to minimize the adverse impact caused by fragments. Tsai et al. demonstrated that performing LAH in the morula stage could promote blastocyst formation by releasing fragments out of ZP [28]. In our earlier research, we demonstrated that drilling the ZP of day 4 embryos with a 10 μm laser beam could greatly increase the BFR of LGCEs [9]. However, it was also observed that some of the holes drilled on ZP were re-occluded later by fragments or blastomeres. This is the rationale behind our study to perform a repeat LAH on day 6. Unlike the primary LAH, we performed a larger incision of more than 30 μm on ZP so that more embryotoxic substances could be discharged as quickly as possible. The incision on ZP was made close to the blastomere with apparent fragments or the isolated blastomere out of the compaction area. It was to minimize the influence on the crucial compaction stage of the embryo [29].

The spatial relationship of fragments or isolated blastomeres in the morula is a major factor to determine the excision type on ZP. The ZP of the majority of embryos can be linearly removed, as shown in Fig. 1a. However, due to spatial limitations, nonlinear drilling such as C-shape mode was occasionally used (Fig. 1b). In cases where it is difficult to avoid blastomeres, we performed LAH that passed through the isolated blastomeres (Fig. 1c). The current consensus holds that these expelled free blastomeres are dysfunctional [30] and will not contribute to the development of the embryos.

In the present study, we observed that all the day 7 blastocysts formed in the two groups originated from the morulae on day 6. According to previous study, the M2 and M3 phase morulae on day 6 had BFR of 42.9% and 63.6%, respectively [28]. In our study, the proportion of day 6 morulae was not significantly different between the two groups, but the morulae that underwent Dual-LAH had a higher BFR on day 7. We believed that performing LAH twice on LGCEs improved the efficiency of harmful substances disposal and improved the culture environment.

It is acknowledged that the presence of fragments in embryos is related to an increased level of ROS and apoptosis, impairing embryonic development. Dual-LAH facilitates the diffusion of ROS out of embryos and reduces intra-blastocyst oxidative stress. Additionally, it also provides an artificial route for nutrients in the culture medium to enter the embryo [31]. Furthermore, a study on mouse embryos revealed that performing LAH on ZP before blastocyst stage altered the expression of numerous genes involved in xenogeneic metabolism pathway, which could lead to an increase in BFR [32].

To further maximize the clinical value of LGCEs through the application of Dual-LAH, advances in the subsequent processes are awaited. First of all, it is more difficult to freeze and thaw the stage 6 blastocysts because they are no longer protected by ZP. Therefore, it is necessary to explore a more secure cryopreservation technique for these embryos. Secondly, the individualized window of implantation is still under exploration. In our center, we routinely transferred blastocysts with delayed development on day 5 post progesterone administration, in accordance with current general consensus [33]. With this timing, according to the limited data from our center, 12 Dual-LAH day 7 blastocysts yielded two live births, while 8 D4-LAH day 7 blastocysts yielded one live birth. We look forward to prospective studies with larger sample size to determine the optimal window of implantation for day 7 blastocysts.

## Conclusion

This study established a new culture method for LGCEs in order to harvest blastocyst on day 7. The result showed that performing Dual-LAH led to a higher BFR and usable blastocyst rate, increasing the cumulative embryo count and chance of pregnancy in one cycle of IVF-ET. It also provides a last hope for couples who have only LGCEs to avoid a futile cycle.

## Data Availability

Document with complete data can be made available upon request to the responsible researcher.
